# Isolation protocol for a COVID-2019 patient requiring emergent surgical intervention: case presentation

**DOI:** 10.1186/s13037-020-00243-9

**Published:** 2020-04-19

**Authors:** Michael S. Firstenberg, Matthew Libby, Michael Ochs, Jennifer Hanna, Julie E. Mangino, Joseph Forrester

**Affiliations:** 1 Cardiothoracic and Vascular Surgery, The Medical Center of Aurora, 1444 S. Potomac Street, Suite 200, Aurora, CO 80012 USA; 2 The Ohio State University Wexner Medical Center, Columbus, OH USA

**Keywords:** COVID-2019, SARS-CoV-2, Infection control, Emergency surgery

## Abstract

**Background:**

The concerns of the highly contagious and morbid nature of Coronavirus Disease-2019 (COVID-2019) have prompted healthcare workers to implement strict droplet and contact isolation precautions. Unfortunately, some patients who may be or presumptively or confirmed as infected with COVID-2019 may also require emergent surgical procedures. As such, given the high-risk for exposure of many healthcare workers involved the complex requirements for appropriate isolation must be adhered to.

**Case presentation:**

We present our experience with a 77-year-old who required emergency cardiac surgery for a presumed acute aortic syndrome in the setting of a presumed, and eventually confirmed, COVID-2019 infection. We outline the necessary steps to maintain strict isolation precautions to limit potential exposure to the surgical Team.

**Conclusions:**

We hereby provide our algorithm for emergent surgical procedures in critically-ill patients with presumptive or confirmed infection with COVID-2019. The insights from this case report can potentially be templated to other facilities in order to uphold high standards of infection prevention and patient safety in surgery during the current COVID-19 pandemic.

## Introduction

The impact of the novel “severe acute respiratory syndrome coronavirus-2” (SARS-CoV-2) and the associated infection, Coronavirus Disease-2019 (COVID-2019), on all aspects of humanity and our healthcare workers cannot be overstated [1]. Nevertheless, as the number of positive cases continue to grow – with the associated morbidity and mortality, it is critical to control further spread via implementation and strict compliance with isolation precautions, as healthcare workers and other patients are at significant risk for exposure [2]. SARS-CoV-2 is a respiratory pathogen that is spread predominately through large droplets and contact (i.e. fomites such as surfaces) transmission [3]. As such, respiratory and contact isolation precautions are paramount in limiting further exposure. Because of the highly contagious nature of SARS-CoV-2 and its case fatality rate, much of which is still under investigation, additional precautions are recommended beyond what is typically used for limiting the spread of routine respiratory pathogens [4]. Unfortunately, as the numbers of cases increase, some of the presumptively infected or confirmed SARS-CoV-2 patients will present with concomitant problems that might require surgical intervention.

Few specific guidelines are available to outline the steps necessary to adequately maintain appropriate isolation precautions in patients who require emergent surgical interventions and who are either confirmed SARS-CoV2 or who are awaiting the results of testing. The purpose of this discussion is to outline the steps our U.S. institution undertook in managing a patient who required emergent cardiac surgery for an acute aortic syndrome who was presumptively infected, and did test positive on post-operative day two. This is particularly important due to reports of the virus still being found on un-sanitized surfaces for up to 17 days [3].

## Case presentation

**Fig. 1:**
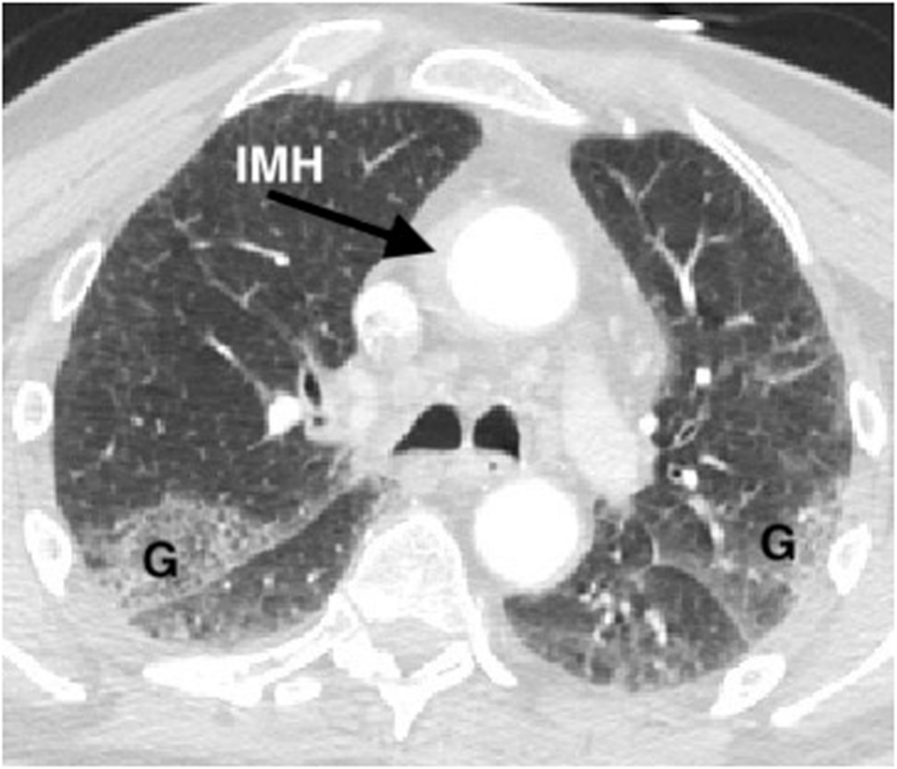
Computed Tomography. Computed tomography (CT) scan of the chest with contrast demonstrates an Intramural Hematoma (IMH) of the ascending thoracic aorta that extended from the aortic root to the proximal aortic arch. In addition, there were patchy peripheral ground-glass (G) opacities un the bilateral upper and lower lobes that have been associated with COVID-2019 infection

Our patient is a 77-year-old retired physician who was reported to be otherwise healthy except for a known hypertension. He experienced a fall at home and presented to a local emergency room with a questionable loss of consciousness and fever. Because of the high rate of community acquired infections in the area of the local emergency room, a complete COVID-2019 workup that included a Computed Tomography (CT) scan of the chest was obtained. The CT scan demonstrated characteristic ground glass opacities which have been associated with COVID-2019. Additionally, there was concern of an intramural hematoma and an acute ascending aortic dissection could not be ruled out. He was transferred to our institution for further management. Due to the concerning CT report and lack of availability of the images, the CT scan was repeated with specific heart-rate gating and contrast bolus administration focusing on his aortic pathology. Again, an ascending aortic hematoma was visualized, and a Type A aortic dissection could not be ruled out (Fig. 1). Given the findings of pulmonary infiltrates, upon admission, he was placed in strict droplet and contact precautions in a negative air-flow Intensive Care Unit (ICU) room as a potential COVID-2019 case. Testing had been obtained prior to transfer. Given his presentation and presumed acute aortic syndrome, and after a multi-disciplinary discussion and shared decision making of the risks and benefits with the patient and his son, it was determined that he should undergo emergent cardiac surgical intervention. Again, because of the risk of exposure, a confirmatory transesophageal echocardiogram was deferred until he was in the operating room. Because of his presumed COVID-2019 infection, we established the following protocol to limit potential HCF spread:

### Preparing for surgery

**Fig. 2:**
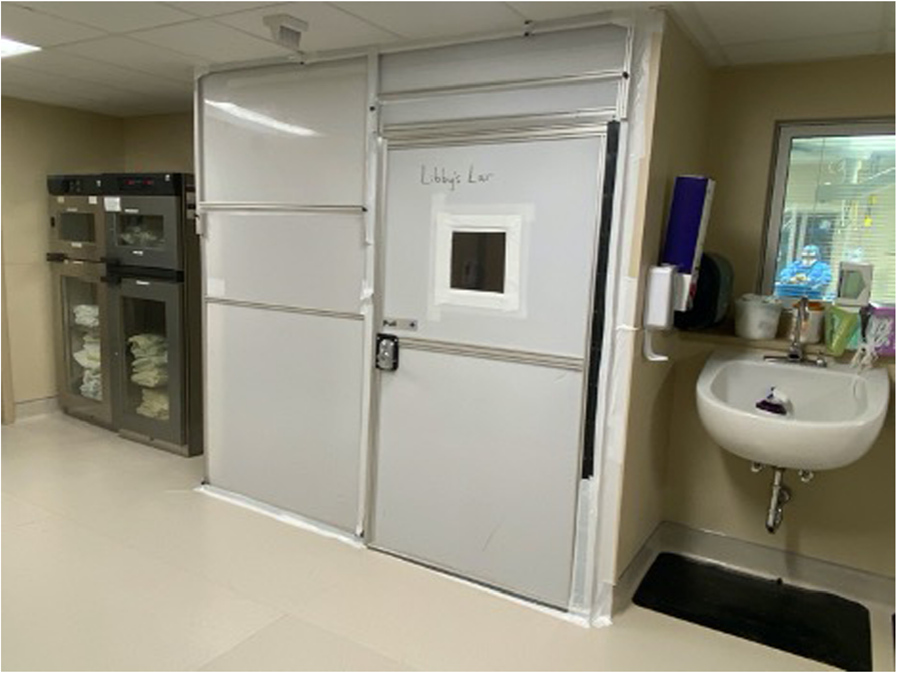
Containment Room. External view of containment room emergently constructed by Hospital Plant Operations to separate the hallway outside of the operating room from the operating room. The inner chamber (approx. 1 × 3 meters wide by length) allowed for passage of supplies into the operating room without directly exposing the external area to the internal operating room. Only one door was allowed to be open at a time and a small table was placed inside to place objects needed by the surgical Team. Dry erase boards were used to communicate via the window

- Written surgical and anesthesia consent was obtained by the operating surgeon and anesthesiologist wearing appropriate personal protective equipment (PPE) that included N-95 mask, face shield, gown, and gloves). All wearable items, including the pen used to sign the consent were disposed of after the consent was signed. The patient was not permitted to touch the consent form.
- All pre-operative labs (as per cardiac surgery routine) were obtained in the ICU and included baseline complete metabolic profile, complete blood count, platelet mapping, thromboelastogram, coagulation profile, and a type and cross.
- Blood from the blood bank was delivered to the Operating Room (OR) prior to the patient arrival to minimize additional opening of the operating room doors. The blood bank was notified that the patient was a “rule-out for COVID-2019”.
- All unnecessary equipment, disposable supplies, paper, which were not in a closed cabinet were removed from the OR.
- Plastic tarps were taped over all equipment and furniture that could not be removed from the OR (e.g., the physician’s desk, supply cabinets, perfusion cabinets, and supply room door.
- Hospital Plant Operations constructed an emergent containment room outside of the OR door (Fig. 2).
- Plant Operations adjusted the pressure in the room to be negative airflow to limit air flow out of the OR.
- Unlike traditional surgical procedures, instrument packs were not opened until the patient was already draped and prepped and the procedure was ready to start.

### Transporting the patient from the ICU to the OR

- The patient was intubated prior to the procedure in the negative airflow ICU by the Attending Intensivist, to avoid potential exposure to the Anesthesia team.
  - Intubation was performed using appropriate precautions as recommended by the Centers for Disease Control (CDC), including N95 mask or PPE, gown, eye protection and hair cover) [5].
- The OR transport team (Attending Anesthesiologist and 2 surgical nurses) were also wearing appropriate PPE.
- The patient was placed on a portable travel ventilator with a High-Efficiency Particulate
  Air (HEPA) filter placed between the endotracheal tube and the circuit and a second HEPA filter between the circuit and ventilator.
- Two members of hospital security escorted the patient and team to the OR to ensure elevator availability, open doors, and to minimize the risk of accidental contact with others during the transport.

### Surgery

- Once in the OR, the patient was moved from the ICU bed to the OR table, and all sheets and gowns were immediately disposed of
- The two HEPA filters remained in place as the patient was connected to the anesthesia circuit.
- Only patient identifier labels were allowed in the OR. The remainder of his chart was available at the front desk of the operating room suites.
- Once inside the OR, all transport monitors, ventilator, and all additional mobile equipment was kept inside the OR.
- Two runners were made available outside of the OR. Only basic supplies were opened in the room for the procedure, and the runners were tasked with obtaining any additional needed supplies for the case. Once obtained, they placed them on a cart in the containment room, and only when the outside door was shut could the operating room door be opened to collect the supplies.
- Communication between those in the room and the supply runners was accomplished via dry erase boards. No additional phones were allowed in the room.
- The procedure was performed using techniques consistent with the standard of care for the procedure. In addition to a surgical gown and gloves, the operating team (e.g., attending surgeon, physician’s assistant, scrub technician, anesthesiologist, perfusionists) wore N95 masks with a face shield or goggles. No breaks or changes in staff were allowed.

### Additional details

#### Blood utilization

- The blood bank did all of the verification of the two units of packed red blood cells that we had in the room, so that the cooler only needed to be opened, if necessary. If the cooler remained shut, the outside could be bleached and returned to the blood bank and the units salvaged. If we had used the blood, all contents of the cooler (insulation, gel pack, ice pack, etc.) would be disposed of within the room, and the cooler was to be wiped down inside and out and then returned to the blood bank.
- Blood was not used during this procedure, but a similar protocol is to be followed for any additional blood products, if needed, including platelets, fresh frozen plasma, and cryoprecipitate.

#### Microbiology

- Specimens were to be placed into a biohazard bag inside the OR and subsequently placed in another biohazard bag in the containment room before being sent to the lab. “Rule-Out for COVID-2019” was written on the specimen label.
- All specimens were transported to the lab and processed in a manner consistent with potentially infectious material obtained during routine surgical procedures.

#### Transporting the patient Back to the ICU

- The previously constructed containment room (Fig. 2) walls/door were taken down to allow for the patient to leave the operating room.
- The ICU team (two nurses, a respiratory therapist, and the Attending Intensivist) came to receive the patient from the OR. They donned appropriate PPE and, travelled with security escort. The OR team (perfusionist, nurses, scrub technician, anesthesiologist, and physician’s assistant) remained in the OR to properly doff PPE.
- The ICU team brought the ventilator the patient would be on in the unit. The HEPA filter on the endotracheal tube was kept in place, and the patient was placed on that ventilator for transport back to the ICU. This saved the circuit from being broken in the ICU if we had we used a transport vent.
- The surgical chest tube was hooked up to a Pleuravac® (Teleflex, Morrisville, NC USA) as per standard surgical practice and left on water seal.

#### Operating room clean-up

- All trash and linen were placed in red biohazard bags. Special care was taken when dismantling and discarding the circuit from the anesthesia machine.
- The room was left empty for overnight as no other cases needed to be scheduled in that room.
- The OR was terminally cleaned, per routine hospital protocol, and an anesthesia tech serviced and cleaned the anesthesia machine.
- Sterile processing was made aware of the COVID-2019 status when the dirty instruments were taken to that department. In addition to the normal PPE that the department used to decontaminate the surgical instruments, the individuals wore an N95 mask.
- Lastly, the ultraviolet light disinfection was set on its normal cycle and run in the OR room to additionally disinfect the room – and the tarps were removed and destroyed.
- All surgical scrub attire was immediately removed by all members of the team for routine cleaning

Upon arrival to the ICU, routine post-cardiac surgery orders were initiated, and the patient was extubated within 6 h to minimal oxygen support. His initial post-operative course was uneventful. On post-operative day #1, surgical lines and catheters were removed, and his chest tube was removed on post-operative day #3. His SARS-CoV2 test result returned as positive on post-operative day #3 and he remains under isolation precautions pending discharge to an appropriate rehabilitation facility. The family did not visit him during his hospitalization due to the concern of COVID-2019 and subsequent anticipated need for self-quarantine, which is now hospital policy as well to not have visitors for suspect or confirmed COVID-2019 patients.

This patient was determined to has had strict source control of this infection at all times, and all providers – including all members of the surgical team – adhered to recommended protocols. All healthcare workers are deemed (per current CDC recommendations) to be at low risk and instructed to “self-monitor for symptoms”; no work restrictions were applied.

As of final submission of this manuscript, post-operative day number 15, no member of the ICU or Surgical Team involved with this patient has developed symptoms or tested positive for COVID-2019; the patient continues to have an uneventful recovery from this cardiac surgery and the SARS-CoV-2 infection.

## Conclusion

Pandemic COVID-2019 remains a substantial global problem. Until appropriate treatments and vaccines are available, limiting exposure is considered the best option for preventing further disease spread, especially among at-risk healthcare providers. Unfortunately, it is a reality that patients who are either infected or have an unknown infectious status might require emergent surgical intervention before testing results are available [6]. Strict adherence to droplet and contact isolation precautions for SARS-CoV-2 must be maintained to minimize the risk to healthcare providers. Hopefully, our protocol will illustrate some of the key steps necessary to safely and appropriately surgically intervene in a critically-ill patient, who is or might be infected with COVID-2019.

## Data Availability

N/A

## Abbreviations

CDC: Centers for Disease Control
COVID-2019: Coronavirus Disease-2019
CT: Computed tomography
HEPA: High-efficiency Particulate Air
ICU: Intensive Care Unit
OR: Operating Room
PPE: Personal Protective Equipment
SARS-CoV-2: Severe Acute Respiratory Syndrome Coronavirus-2
